# Pulse high-volume haemofiltration for treatment of severe sepsis: effects on hemodynamics and survival

**DOI:** 10.1186/cc3529

**Published:** 2005-04-28

**Authors:** Ranistha Ratanarat, Alessandra Brendolan, Pasquale Piccinni, Maurizio Dan, Gabriella Salvatori, Zaccaria Ricci, Claudio Ronco

**Affiliations:** 1 Fellow, Department of Nephrology, Dialysis and Transplantation, St Bortolo Hospital, Vicenza, Italy, and Instructor, Department of Medicine, Faculty of Medicine Siriraj Hospital, Mahidol University, Bangkok, Thailand; 2 Nephrologist and Consultant in Nephrology, Department of Nephrology, Dialysis and Transplantation, St Bortolo Hospital, Vicenza, Italy; 3 Head of Department, Department of Anesthesia and Intensive Care, St Bortolo Hospital, Vicenza, Italy; 4 Fellow, Department of Nephrology, Dialysis and Transplantation, St Bortolo Hospital, Vicenza, Italy; 5 Professor and Head of Department, Department of Nephrology, Dialysis and Transplantation, St Bortolo Hospital, Vicenza, Italy

## Abstract

**Introduction:**

Severe sepsis is the leading cause of mortality in critically ill patients. Abnormal concentrations of inflammatory mediators appear to be involved in the pathogenesis of sepsis. Based on the humoral theory of sepsis, a potential therapeutic approach involves high-volume haemofiltration (HVHF), which has exhibited beneficial effects in severe sepsis, improving haemodynamics and unselectively removing proinflammatory and anti-inflammatory mediators. However, concerns have been expressed about the feasibility and costs of continuous HVHF. Here we evaluate a new modality, namely pulse HVHF (PHVHF; 24-hour schedule: HVHF 85 ml/kg per hour for 6–8 hours followed by continuous venovenous haemofiltration 35 ml/kg per hour for 16–18 hours).

**Method:**

Fifteen critically ill patients (seven male; mean Acute Physiology and Chronic Health Evaluation [APACHE] II score 31.2, mean Simplified Acute Physiology Score [SAPS] II 62, and mean Sequential Organ Failure Assessment 14.2) with severe sepsis underwent daily PHVHF. We measured changes in haemodynamic variables and evaluated the dose of noradrenaline required to maintain mean arterial pressure above 70 mmHg during and after pulse therapy at 6 and 12 hours. PHVHF was performed with 250 ml/min blood flow rate. The bicarbonate-based replacement fluid was used at a 1:1 ratio in simultaneous pre-dilution and post-dilution.

**Results:**

No treatment was prematurely discontinued. Haemodynamics were improved by PHVHF, allowing a significant reduction in noradrenaline dose during and at the end of the PHVHF session; this reduction was maintained at 6 and 12 hours after pulse treatment (*P *= 0.001). There was also an improvement in systolic blood pressure (*P *= 0.04). There were no changes in temperature, cardiac index, oxygenation, arterial pH or urine output during the period of observation. The mean daily Kt/V was 1.92. Predicted mortality rates were 72% (based on APACHE II score) and 68% (based on SAPS II score), and the observed 28-day mortality was 47%.

**Conclusion:**

PHVHF is a feasible modality and improves haemodynamics both during and after therapy. It may be a beneficial adjuvant treatment for severe sepsis/septic shock in terms of patient survival, and it represents a compromise between continuous renal replacement therapy and HVHF.

## Introduction

Severe sepsis represents the leading cause of mortality and morbidity in critically ill patients worldwide. The sepsis syndrome is associated with an overwhelming, systemic overflow of proinflammatory and anti-inflammatory mediators, which leads to generalized endothelial damage, multiple organ failure and altered cellular immunological responsiveness. Although our understanding of the complex pathophysiological alterations that occur in severe sepsis and septic shock has increased greatly as a result of recent clinical and preclinical studies, mortality associated with the disorder remains unacceptably high, ranging from 30% to 50% [1-4].

The cornerstone of therapy continues to be early recognition, prompt initiation of effective antibiotic therapy, source control, and goal-directed haemodynamic, ventilatory and metabolic support as necessary. To date, attempts to improve survival with innovative, predominantly anti-inflammatory therapeutic strategies have been disappointing, with the exception of physiological doses of corticosteroid replacement therapy [5,6] and activated protein C (drotrecogin alfa [activated]) [7] in selected patients.

'Renal dose' haemofiltration rate of 2000 ml/hour has successfully been used to treat acute renal failure for years [8]. This dose suffices for renal replacement therapy and can remove inflammatory mediators; however, it does not alter plasma levels of these mediators, suggesting that its ability to clear inflammatory mediators is suboptimal [9]. This was reflected in one study [10] by failure to demonstrate an improvement in organ dysfunction and survival. Hence, the indication for its use in septic patients was abandoned, beyond its function to provide renal support in the presence of renal dysfunction [11]. However, the theory that underpins increasing plasma water exchange or higher dose haemofiltration seems reasonable.

Ronco and coworkers [12] demonstrated survival benefits by increasing the haemofiltration dose (35 ml/kg per hour) beyond the conventional renal dose (20 ml/kg per hour), but no further benefit was achieved, even at higher doses (45 ml/kg per hour), in the overall studied population. Nevertheless, there was an improvement in survival at the highest haemofiltration doses in that study for the subset of patients with sepsis. Additionally, benefits have been demonstrated in several animal models of sepsis. Improvements in cardiac function and haemodynamics were replicated in these animal studies using ultrafiltration (UF) rates up to 120 ml/kg per hour [13-16]. Septic dose haemofiltration, or high-volume haemofiltration (HVHF), was thus conceived and applied in human sepsis. Findings of improvements in haemodynamics with decreased vasopressor requirements [17-19] and trends toward improved survival [19,20] are evidence that HVHF may be efficacious. Because HVHF technique requires high blood flows, tight UF control and large amounts of expensive sterile fluids, we proposed a new technique, namely 'pulse HVHF' (PHVHF) [21,22]. PHVHF is application of HVHF for short periods (up to 6–8 hours/day), providing intense plasma water exchange, followed by conventional continuous venovenous haemofiltration (CVVH).

We hypothesized that daily 'PHVHF' may have beneficial effects in severe sepsis by unselectively removing of proinflammatory and anti-inflammatory mediators, and hence improving patient outcomes. The present study evaluates the feasibility of PHVHF and the effect of this treatment on haemodynamics, oxygenation and 28-day all-cause mortality.

## Materials and methods

This is a prospective interventional study conducted in the intensive care unit (ICU) of St. Bortolo Hospital, Vicenza, Italy. Fifteen patients with severe sepsis receiving continuous renal replacement therapy (CRRT) were enrolled in the study. Patients were included in the study if they had severe sepsis or septic shock, as defined using the criteria reported by Bone and coworkers [23], and if they fulfilled one of the previously reported criteria for initiating renal replacement therapy in critically ill patients [24]. Exclusion criteria were age less than 18 years, death imminent within 24 hours, and very high weight (>140 kg). All patients were treated using the same, recently developed management guideline for severe sepsis and septic shock [25]. All except one patient were receiving mechanical ventilation because of respiratory failure. Broad spectrum antibiotics were given to all patients and were altered according to blood culture and sensitivity findings.

Eight out of 15 patients received activated protein C (drotrecogin alfa [activated]). The drug was not used in seven patients: one had underlying ruptured abdominal aortic aneurysm; the second was admitted because of multiple fractures and severe head trauma; the third had an Acute Physiology and Chronic Health Evaluation (APACHE) II score less than 25 at admission; and the remaining four had severe thrombocytopenia (<15,000/mm^3^) and/or impaired coagulation (international normalized ratio >3.0). The use of activated protein C (drotrecogin alfa [activated]) in approximately 50% of the patients included might therefore have contributed to any improved outcome identified. Clinical data are summarized in Table 1.

The APACHE II score, Simplified Acute Physiology Score (SAPS) II, and Sequential Organ Failure Assessment score were calculated from physiological measurements obtained during the first 24 hours of ICU admission. Expected mortality rates for APACHE II and SAPS II scores were computed using the logistic regression calculations suggested in the original reports [26,27]. The study protocol was approved by the hospital ethics committee.

### Description of pulse high-volume haemofiltration technique

PHVHF was performed using a multifiltrate CRRT machine (Fresenious Medical Care, Bad Hamburg, Germany). This recently designed machine provides high-precision scales (equipped with software for online continuous testing and high capacity) and powerful heating systems for maintaining the large volumes of infusion solution at sufficiently high temperature.

Vascular access was obtained with 14-Fr central venous haemodialysis catheter. Blood flow rates of 250–300 ml/min, as permitted by the access, were used to achieve a filtration fraction of 20–25% and to prevent premature clotting of extracorporeal circuit.

PHVHF was performed using a UF rate of 85 ml/kg per hour for 6 hours/day followed by standard continuous venovenous haemofiltration (CVVH; UF rate 35 ml/kg per hour) for 18 hours, resulting in a cumulative dose of approximately 48 ml/kg per hour. Treatments were given on a daily basis, and were terminated if the patient died or if the physician considered the septic process to have ended and the patient's clinical parameters improved.

Commercially available bicarbonate-buffered replacement fluid containing sodium 142 mmol/l, potassium 2 mmol/l, chloride 113.5 mmol/l, bicarbonate 32 mmol/l and calcium 1.75 mmol/l (Bi-intensive; B-Braun, Bologna, Italy) was used at a ratio of 1:1 in simultaneous pre-dilution and post-dilution. Additional potassium and phosphate were administered intravenously to prevent hypokalaemia and hypophosphataemia. A highly biocompatible synthetic membrane with surface area of 1.8–2 m^2 ^was also utilized. Anticoagulation was initiated with 1000–2000 IU bolus injection of heparin followed by an infusion of 250–500 IU/hour. Net fluid removal was set according to the patient's condition and clinical need.

### Measurements

Haemodynamic monitoring was done using a thermodilution pulmonary artery catheter with continuous cardiac output monitoring (Vigilance; Edwards Lifesciences, Irvine, CA, USA). A radial or a femoral arterial catheter was used to measure blood pressure and obtain arterial blood for blood gas analysis. Systolic blood pressure, mean arterial pressure (MAP), body temperature, heart rate, cardiac index and noradrenaline (norepinephrine) dose required to maintain MAP above 70 mmHg were measured immediately before PHVHF, mid-PHVHF, immediately after PHVHF, and 6 hours and 12 hours after completion of the PHVHF session. The bedside nurse was instructed to maintain MAP above 70 mmHg by adjusting the dose of noradrenaline infused. pH, partial oxygen tension and bicarbonate were measured using a clinical blood gas analyzer (Rapidpoint 400; Bayer HealthCare, Newbury, UK) at similar time intervals.

Blood samples were also collected at immediately before initiation of treatment, immediately on discontinuation of PHVHF and 12 hours after the session had ended, in order to measure blood urea nitrogen, creatinine and electrolytes. Observed mortality was recorded during the day on which patients received PHVHF and at 28 days.

### Data analysis

One-sample Kolmogorov–Smirnov test was utilized to assess whether the distribution of haemodynamic and metabolic variables were normal. Normally distributed data are presented as means ± standard deviation, and differences of serially measured variables were analyzed using analysis of variance for repeated measurements with Bonferroni correction. For non-normally distributed variables, results are reported as medians with 25th to 75th percentile range, and Friedman's two-way analysis of varience with *post hoc *Wilcoxon signed rank test was used to identify whether changes had occurred over time. Comparison between expected mortality (based on APACHE II and SAP II scores) and observed mortality was done using the standardized ratio and 95% confidence interval calculated by dividing the observed by expected mortality [28]. *P *< 0.05 was considered statistically significant.

## Results

### Patient outcomes

Of the 15 patients enrolled, 50 PHVHF treatments were performed on a daily basis. The mean number of treatments per patient was 3.4 (1–9). No treatment was prematurely discontinued because of extracorporeal circuit clotting or high pressure problems. Demographic data are presented in Table 1. The observed patient hospital mortality was 46.7%, as compared with a rate of 72% predicted by APACHE II and 68% predicted by SAPS II severity scores. Hospital mortality ratios (95% confidence interval) [28] were 0.65 (0.48–0.87) and 0.69 (0.51–0.92), as compared with the expected mortality calculated from APACHE II and SAPS II scores, respectively.

With respect to causes of death, one patient died from acute myocardial infarction with cardiogenic shock during day 7 of ICU admission. The second patient, with acute endocarditis, underwent PHVHF for 2 days and all vasopressors (noradrenaline, adrenaline and dopamine) were discontinued on day 3.

Unfortunately, the patient had cardiogenic shock from a ruptured aortic valve on day 7 and died on day 9 after admission. The third patient died because her underlying disease was multiple myeloma grade IIIb, which did not respond to chemotherapy, and the physician decided to withhold the treatment, in accordance with hospital policy, on day 9 after admission. Only the remaining four patients died from refractory septic shock.

Table 2 summarizes baseline demographic and physiological parameters, stratifying patients by whether they were alive at 28 days. Before initiation of PHVHF there were no significant differences between survivors and nonsurvivors at 28 days with respect to age, body weight, MAP, cardiac index, oxygenation, severity scores (APACHE II, SAPS II and Sequential Organ Failure Assessment) and number of organ failures. Interestingly, the mean number of PHVHF treatments per patient was significantly higher in the group of survivors (4.8 ± 2.7) than in the nonsurvivor group (1.9 ± 0.7; *P *= 0.02).

### Haemodynamic outcomes

All patients except three received noradrenaline at the start of PHVHF treatment, with a median dose of 48 μg/min (Table 3). In fact, dopamine is generally the first-choice vasoactive/inotropic agent in our unit; however, once the dopamine infusion has exceeded 10 μg/kg per min or low systemic vascular resistance is identified by pulmonary artery catheter, our policy is to initiate noradrenaline and taper dopamine. As a result, noradrenaline was the sole vasoactive agent in one patient only. The remaining three patients were receiving dopamine with or without dobutamine at the initiation of PHVHF therapy. The median number of concurrently administered vasopressors per patient before PHVHF was 2, and this did not change after PHVHF. No patients developed threatening hypotension during pulse therapy, and none needed *de novo *institution of vasopressors during this treatment.

**Figure 1:**
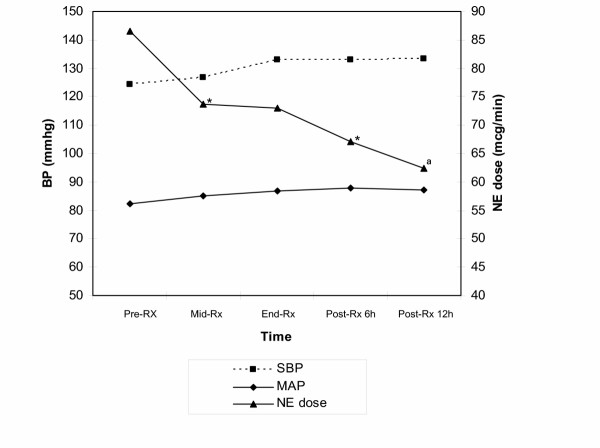
Haemodynamic variables. Variables were recorded during the pulse high-volume haemofiltration (PHVHF) session, and 6 hours and 12 hours after completion of the session. Noradrenaline (norepinephrine [NE]) requirement decreased significantly during treatment, and this reduction persisted at 6 hours and 12 hours after treatment (*P *= 0.0001). *P < 0.05 and ^a^*P *< 0.01 for difference between pre-PHVHF and other measures over time. Sytolic blood pressure (SBP) increased significantly during treatment, and this was maintained 6 hours and 12 hours after treatment (*P *= 0.04). All reported values are means.

Haemodynamic changes are shown in Table 3. MAP before PHVHF was 82 ± 18 mmHg, after PHVHF it was 87 ± 18 mmHg, and 12 hours after PHVHF it was 87 ± 22 mmHg (*P *= 0.2). However, systolic blood pressure increased significantly over time (pre-PHVHF 124 ± 26 mmHg, mid-PHVHF 127 ± 22 mmHg, post-PHVHF 133 ± 25 mmHg, 6 hours after PHVHF 133 ± 24 mmHg, and 12 hours after PHVHF 133 ± 26 mmHg; *P *= 0.04). As expected, MAP and cardiac index did not change significantly over time during PHVHF and after treatment, and MAP was maintained at the target levels in accordance with the study protocol (Table 3). The dose of noradrenaline required for maintenance of target MAP decreased significantly by the mid-point of the PHVHF session, and this decrease was maintained at 6 and 12 hours after treatment (*P *= 0.001; Table 3 and Fig. 1).

By setting the temperature of the replacement fluid at around 38.5–39°C, body temperature was constant during pulse treatment (Table 3). Positive fluid balance on the day before PHVHF (1374 ± 2618 ml/day) was not different from that during the day on which patients underwent PHVHF (1514 ± 2548 ml/day; *P *= 0.9). Oxygenation (arterial oxygen tension/fractional inspired oxygen ratio) did not change over time.

### Solute control and renal outcomes

Seven out of eight survivors underwent CVVH after the termination of daily PHVHF treatments because of renal failure. In one survivor renal function recovered by the time of cessation of daily PHVHF. All except two kidney transplant recipients (in whom the graft was lost because of septic shock) could be withdrawn from renal replacement therapy and had complete renal recovery.

Four nonsurvivors at 28 days with refractory septic shock died while they were still receiving daily PHVHF. As mentioned above, three nonsurvivors died for reasons other than septic shock and were treated with CVVH following improvement in their haemodynamic parameters and cessation of PHVHF.

Solutes and acid base status before and after PHVHF are presented in Table 4. Daily Kt/V was 1.92 ± 0.29. As expected, serum blood urea nitrogen and creatinine levels diminished greatly after pulse treatment (*P *< 0.0001; Table 4). Daily urine output on the day before treatment (median 310 ml, range 75–1916 ml) did not differ from that on the day of initiation of PHVHF treatment (median 268 ml, range 77–1905 ml).

## Discussion

The sepsis syndrome is associated with an overwhelming, systemic overflow of proinflammatory and anti-inflammatory mediators, which leads to generalized endothelial damage, multiple organ failure and altered cellular immunological responsiveness. The complex inflammatory network involved is redundant, synergistic and acts like a cascade. It includes mediators with autocrine and paracrine actions, as well as cellular and intracellular components. A large number of proinflammatory mediators, including tumour necrosis factor-α, interleukin-1, interleukin-6, platelet-activating factor and nitric oxide, play important roles in the cascade, but attempts to improve survival in human trials using innovative, predominantly anti-inflammatory therapeutic strategies have been disappointing [29]. Almost paralleling the surge in proinflammatory mediators, there is a rise in anti-inflammatory substances, resulting in induction of a state of immunoparalysis or monocyte hyporesponsiveness [30]. Both proinflammatory and anti-inflammatory factors become upregulated and interact with each other, leading to various rises in mediator levels that change over time. Neither therapies directed at single mediators nor single-dose interventions therefore seem appropriate, in part because of a discrepancy between the biological timing of the syndrome and the clinical timing of symptoms.

CRRT has made extracorporeal treatment possible in septic patients even when they are haemodynamically unstable; such treatment is given to balance hypercatabolism and fluid overload. In addition, 'high volume' and convective modalities have the advantage of removing higher molecular weight substances, which include many inflammatory mediators. Multiple animal studies [13-16] have shown a beneficial effect of HVHF on survival in endotoxaemic models. Recent studies in humans [17-19] have demonstrated that HVHF improves haemodyamics, with decreased vasopressor requirements.

A daily PHVHF regimen was utilized as the intervention in the present study for the following reasons. First, the very high UF volume requires very close surveillance, which is difficult to maintain over 24 hours. Second, solute kinetics may render high volumes useless after a few hours because of saturation of membrane adsorption [17,31]. Third, standard CVVH (UF rate 35 ml/kg per hour) may help to maintain the effect of pulse therapy and prevent post-treatment rebound from sudden changes. Instead of using a fixed dose (i.e. UF rate 6 l/hour), we applied a dose of 85 ml/kg per hour during pulse treatment because body size is the main predictor of patient outcome [12,18]. Additionally, 'continuous' removal of soluble mediators may be the most logical and best approach to a complex and lengthy process such as sepsis; we therefore performed PHVHF on a daily basis and terminated treatment when haemodynamic variables improved. We hypothesized that beneficial haemodynamic effects would be achieved during PHVHF, and that these effects would be perpetuated after cessation of the pulse treatment by standard CVVH. We also hypothesized that they would be accompanied by improvement in oxygenation and reduction in mortality. The present pilot study in patients with severe sepsis/septic shock was conducted to test our hypotheses.

Overall, PHVHF was well tolerated by critically ill patients and appeared to offer many of the benefits conferred by continuous HVHF [19] while avoiding its drawbacks. Six to eight hours PHVHF during the daytime was widely accepted by the ICU nursing staff because it reduced the labour intensity of the protocol during the night shift. No treatment was prematurely discontinued because of extracorporeal circuit clotting or high pressure problems. It appears that PHVHF is a feasible modality and can safely be performed on a daily and prolonged basis. The greatest duration of treatment in any patient our study was 9 days. The most clinically relevant finding that emerged from our investigation is that adjuvant PHVHF in septic shock patients is associated with improvement in haemodynamic parameters (Fig. 1), permitting a significant reduction in vasopressor requirements as soon as halfway through and at the end of the PHVHF session, and this was maintained at 6 hours and 12 hours after treatment. The haemodynamic benefits of short-term HVHF regimens were recently demonstrated by Cole and coworkers [17] (UF rate 6 l/hour, duration 8 hours) and Honore and colleagues [18] (UF rate 8.75 l/hour, duration 4 hours). We proved that this beneficial hemodynamic effect can be maintained after HVHF by continuing with standard dose CVVH (UF rate 35 ml/kg per hour). Indeed, in practice our regimen could be adjusted on the basis of the individual patient's clinical response.

Several mechanisms are potentially responsible for the reduced need for pressor therapy with PHVHF. For mediator-independent factors, we were unable to demonstrate any differences in body temperature and arterial pH before and after PHVHF, including 12 hours after treatment. It is clear that cooling-induced vasoconstriction and correction of severe acidosis cannot account for this positive haemodynamic effect. The daily fluid balance on the day before initiation of PHVHF and that on the day of intervention were similar. Based on these findings, we argue that PHVHF permits continuous removal of soluble vasodilatory mediators or molecules identified in sepsis by either convection or adsorption, resulting in reduction in vasopressor requirements.

Unlike recent studies conducted by Honore and coworkers [18] and Joannes-Boyau and colleagues [19], we was unable to demonstrate any benefit of HVHF on cardiac index. The possible explanation for this is that we recruited patient at an earlier time point in septic shock (i.e. during hyperdynamic state); the mean cardiac index of our patients was 3.4 l/min per m^2^, whereas those in the other two studies were less (2.0 l/min per m^2 ^[18] and 2.9 l/min per m^2 ^[19]). The aim of haemodynamic support in our sepsis patients was to maintain condiac index at 2.5 l/min per m^2 ^or above because the studies that attempted to maintain a supraphysiologic cardiac index of above 4.0 to 4.5 l/min per m^2 ^have not shown consistent benefit [32,33]. Interestingly, this indicates that the improved haemodynamics and decreased vasopressor requirement conferred by daily PHVHF are clinically significant even during hyperdynamic septic shock.

Although it is beyond the scope of this report to provide a full comparison of mortality rates between standard sepsis treatment and such treatment combined with PHVHF, it appears that PHVHF may have beneficial immunomodulatory effects with prolonged daily use, especially with respect to patient outcome. The 28-day all-cause mortality was 47%, as compared with 72% as predicted by APACHE II and 68% as predicted by SAPS II severity scores. This is consistent with the findings of another study [19], in which 96 hours of continuous HVHF was given to patients with septic shock (46% observed and 70% predicted mortality rate). In fact, of the seven deaths at 28 days in our study, only four were attributable to refractory septic shock. How long would it take for a clinically relevant benefit to manifest? Tailoring our daily PHVHF regimen according to clinical response should permit sufficient duration of HVHF. In addition, because absolute or relative contraindications were met in seven patients, only the remaining eight patients in the present study received activated protein C (drotrecogin alfa [activated]) – a drug that has shown the benefit in terms of 28-day mortality in recent trials [7]. However, we can state that PHVHF is feasible and, as a treatment for severe sepsis/septic shock, can affect physiological end-points. In terms of mortality, the only way to demonstrate the effect of PHVHF in this population is to conduct a prospective, randomized, controlled study on a larger scale. Nevertheless, we can hypothesize that the use of activated protein C (drotrecogin alfa [activated]) in 50% of the population might have contributed to the improved outcome. If so, then the combination of activated protein C (drotrecogin alfa [activated]) and PHVHF might be particularly useful.

The present study is limited by the fact that the population was highly heterogeneous, relatively small and reflective of patients seen in a single center. We did not measure mediator levels in plasma and in the UF over time, which might have helped to explain the mechanisms of mediator removal. However, the nonselective, simultaneous removal of different mediators demonstrated by a reduction of the circulating cytokines or an increase their levels in the UF may not necessarily implicate as the gold standard of blood purification for sepsis patients. A more effective strategy would be to attempt to influence the functional responses of cells that are implicated in the pathogenesis of sepsis. Such approaches are under evaluation, and findings reported in a preliminary paper [21] are encouraging. Also, we did not evaluate removal of sedative drugs with vasodilatory effect, such as midazolam and sufentanil. For ethical reasons, we could not conduct the trial in sepsis patients who did not have acute renal failure. In the context of acute renal failure in sepsis, it is clear that metabolic compounds partly accumulate as a consequence of the loss of renal function. Uraemic toxins rapidly accumulate in tissues and plasma, and they may be responsible for the immune dysregulation associated with sepsis.

## Conclusion

In summary, PHVHF appears to be feasible and is a promising technique for the treatment of severe sepsis. We demonstrated a clinically and statistically significant beneficial effect of this therapy on vasopressor requirements during treatment and after therapy. It may be a beneficial adjuvant treatment for severe sepsis/septic shock in terms of patient survival, and it represents a compromise between CRRT and HVHF. Further confirmation is required in large, properly designed clinical trials to establish the benefit of PHVHF.

## Key messages

• 	PHVHF represents a feasible compromise between CRRT and HVHF, in which HVHF is applied for short periods of up to 6–8 hours/day and followed by standard dose CVVH.

• 	PHVHF, when applied in patients with septic shock/severe sepsis, can achieve beneficial effects on vasopressor requirements.

• 	PHVHF applied on the daily basis and tailored according to clinical response may represent a beneficial adjuvant treatment for severe sepsis/septic shock in terms of patient survival.

## Abbreviations

APACHE = Acute Physiology and Chronic Health Evaluation; CRRT = continuous renal replacement therapy; CVVH = continuous venovenous haemofiltration; HVHF = high-volume haemofiltration; ICU = intensive care unit; MAP = mean arterial pressure; PHVHF = pulse high-volume haemofiltration; SAPS = Simplified Acute Physiology Score; UF = ultrafiltration;

## Competing interests

The author(s) declare that they have no competing interests.

## Authors' contributions

RR conducted the study, collected data, performed statistic analysis and drafted the manuscript. AB and ZR conducted the intervention in the study. PP and MD carried out the haemodynamic measurements. GS helped to collect the data. CR conceived the study, participated in its design and helped to draft the manuscript. All authors read and approved the final manuscript.

## Figures and Tables

**Table 1:** Clinical features of patients with septic shock/severe sepsis treated with pulse high volume hemofiltration

Age (years)/sex/body weight (kg)	Number of treatments	Diagnosis	Microbiology	Number of organ failures	APACHE II score^a^	SAPS II score^a^	SOFA score	28-day survival
66/M/77	1	CHF, septic shock	Negative	4	35 (83%)	79 (92%)	14	D
62/M/70	2	Lobar pneumonia	Negative	4	27 (61%)	53 (53%)	11	D
77/M/70	2	Ruptured abdomonal aortic aneurysm, pancreatitis	Nonfermentative Gram-negative bacilli	4	32 (76%)	53 (53%)	14	D
37/M/87	5	Necrotizing fasciitis	Negative	5	29 (67%)	58 (64%)	17	A
69/F/68	3	Kidney transplant, disseminated candidiasis, septicaemia (uncertain source)	*Candida glabrata*, coagulase-negative *Staphylococcus*	4	34 (81%)	86 (95%)	13	A
54/M/80	2	Bronchopneumonia	Negative	3	23 (46%)	46 (37%)	12	A
54/F/45	2	Myelodysplasia, acute endocarditis	*Staphylococcus aureus*, *Escherichia coli*	5	29 (67%)	55 (58%)	17	D
58/F/65	3	Obstructive uropathy, pyelonephritis	*Escherichia coli*	4	28 (64%)	46 (37%)	15	A
64/M/80	1	Exfoliative dermatitis, erysipilas	Haemolytic *Streptococcus *group A	4	39 (90%)	82 (94%)	16	D
74/F/90	2	Nosocomial pneumonia, catheter-related sepsis	*Pseudomonas aeruginosa*, coagulase-negative *Staphylococcus*	4	33 (79%)	61 (70%)	14	A
43/F/63	6	Kidney transplant, disseminated candidiasis, UTI	*Escherichia coli*, *Candida albicans*	3	26 (57%)	32 (42%)	11	A
33/M/85	3	Multiple trauma, infected wound	Coagulase-negative *Staphylococcus*	5	31 (73%)	70 (84%)	13	D
69/F/82	2	Multiple myeloma, peritonitis	Nonfermentative Gram-negative bacilli	4	33 (79%)	74 (88%)	14	D
44/F/83	8	Kidney transplant, septicaemia (uncertain source)	*Pseudomonas aeruginosa*, *Enterococcus faecalis*	4	36 (85%)	68 (81%)	16	A
59/F/63	9	Rheumatoid arthritis, pneumonia	Streptococcal pneumonia	5	33 (79%)	67 (80%)	16	A

^a^Shown in parentheses is the predicted chance of hospital mortality. A, alive; APACHE, Acute Physiology and Chronic Health Evaluation score; CHF, congestive heart failure; D, died; SAPS, Simplified Acute Physiology Score; SOFA, Sequential Organ Failure Assessment; UTI, urinary tract infection.

**Table 2:** Baseline demograpic and physiological variables stratified by outcome (28-day survival)

Variables	Survivor (*n *= 8)	Nonsurvivor (*n *= 7)	*P*
Age (years)	55 ± 13	61 ± 14	NS
Body weight (kg)	75 ± 11	73 ± 13	NS
SBP (mmHg)	98 ± 20	120 ± 32	NS
MAP (mmHg)	68 ± 12	72 ± 13	NS
CI (l/min per m^2^)	4.1 ± 1.1	2.7 ± 1.0	NS
PaO_2_/FiO_2 _ratio	216 ± 99	172 ± 49	NS
APACHE II score	30.3 ± 4.5	32.2 ± 3.9	NS
SAPS II score	58.0 ± 16.6	66.6 ± 12.7	NS
SOFA score	14.3 ± 2.1	14.1 ± 2.0	NS
Number of organ failures	4.0 ± 0.8	4.3 ± 0.5	NS
Number of PHVHF treatments	4.8 ± 2.7	1.9 ± 0.7	0.02

Values are expressed as mean ± standard deviation. APACHE, Acute Physiology and Chronic Health Evaluation score; CI, cardiac index; MAP, mean arterial pressure; PaO_2_/FiO_2_, arterial oxygen tension/fractional inspired oxygen; PHVHF, pulse high-volume haemofiltration; SAPS, Simplified Acute Physiology Score; SBP, systolic blood pressure; SOFA, Sequential Organ Failure Assessment.

**Table 3:** Effects of pulse high-volume haemofiltration on haemodynamic variables

Variables	Pre-PHVHF	Mid-PHVHF	End-PHVHF	6 hours after PHVHF	12 hours after PHVHF	*P*
Noradrenaline Dose (μg/min)	48 (0–114)	40 (0–97)*	40 (0–93)	40 (0–69)*	33 (0–67)**	0.001
SBP (mmHg)	124.32 ± 25.63	126.64 ± 22.10	133.00 ± 24.55	133.06 ± 23.88	133.16 ± 26.15	0.04
MAP (mmHg)	82.16 ± 18.31	85.02 ± 18.82	86.88 ± 17.56	87.76 ± 20.65	87.26 ± 22.05	NS
CI (l/min per m^2^)	3.4 ± 1.1	3.4 ± 1.2	3.5 ± 1.0	3.5 ± 1.1	3.5 ± 1.2	NS
HR (beats/min)	97.28 ± 25.53	99.62 ± 22.94	100.06 ± 21.79	99.94 ± 20.71	95.62 ± 20.66	0.04
Temperature (°C)	36.7 ± 1.0	36.8 ± 0.8	36.8 ± 0.8	36.9 ± 0.8	36.7 ± 0.9	NS
PaO_2_/FiO_2_	230.9 ± 109.1	232.8 ± 104.4	243.0 ± 105.6	230.2 ± 109.9	234.6 ± 106.4	NS

Normally distributed values are reported as mean ± standard deviation, and the statistical test used was analysis of variance for repeated measurements. Non-normally distributed values are reported as median (25th to 75th percentile), and *P *value was determined using Friedman's two-way analysis of varience with post-hoc Wilcoxon signed rank test. *P < 0.05, **P < 0.01 versus baseline. HR, heart rate; CI, cardiac index; MAP, mean arterial pressure; PaO_2_/FiO_2_, arterial oxygen tension/fractional inspired oxygen; PHVHF, pulse high-volume haemofiltration; SBP, systolic blood pressure.

**Table 4:** Effects of pulse high-volume haemofiltration on metabolic variables

Variables	Pre-PHVHF	End-PHVHF	12 hours after PHVHF	*P*
Blood urea nitrogen (mg/dl)	102.5 (80.5–150.5)	86.0 (68.5–109.0)*	94.0 (69.0–138.0)*	<0.0001
Creatinine (mg/dl)	2.5 (1.4–3.5)	1.8 (1.2–2.9)*	1.9 (1.2–2.8)*	<0.0001
pH	7.38 (7.34–7.45)	7.40 (7.35–7.46)	7.39 (7.33–7.45)	NS
HCO_3_^- ^(mmol/l)	23.9 (21.3–25.9)	24.0 (22.4–25.4)	24.0 (22.1–25.7)	NS

Reported values are median (25th to 75th percentiles); *P *values determined using Friedman's two-way analysis of varience with post-hoc Wilcoxon signed rank test. *P < 0.0001 versus baseline. PHVHF, pulse high volume haemofiltration.
